# Cytotoxic lesions of the corpus callosum after COVID-19 vaccination

**DOI:** 10.1007/s00234-022-03010-y

**Published:** 2022-07-09

**Authors:** Hiroya Ohara, Hironori Shimizu, Takehito Kasamatsu, Akihiro Kajita, Kenji Uno, Khin Wee Lai, Balachandar Vellingiri, Kazuma Sugie, Masako Kinoshita

**Affiliations:** 1 Department of Neurology, Minaminara General Medical Center, Yoshino, Nara, Japan; 2 Department of Neurology, Nara Medical University School of Medicine, Kashihara, Nara, Japan; 3 Department of Infection, Minaminara General Medical Center, Yoshino, Nara, Japan; 4 Department of Biomedical Engineering, Faculty of Engineering, University of Malaya, Kuala Lumpur, Malaysia; 5 Department of Human Genetics and Molecular Biology, Bharathiar University, Coimbatore, Tamil Nadu India; 6 Department of Neurology, National Hospital Organization Utano National Hospital, 8 Ondoyama-cho, Narutaki, Ukyo-ku, Kyoto, 616-8255 Japan

**Keywords:** Coronavirus disease 2019, COVID-19, Cytotoxic lesions of the corpus callosum, Mild encephalitis/encephalopathy with reversible splenial lesion, COVID-19 mRNA vaccine

## Abstract

A 23-year-old previously healthy man (Patient 1) and a 33-year-old woman with a past history of depression (Patient 2) developed neurological symptoms approximately 1 week after receipt of the first COVID-19 mRNA vaccination and deteriorated over the next week. Patient 1 reported nausea, headache, a high fever, and retrograde amnesia. Patient 2 reported visual disturbance, headache, dysarthria, a left forearm tremor, dysesthesia of the mouth and distal limbs, and visual agnosia. PCR test results for SARS-CoV-2 were negative. Complete blood cell count, biochemistry, and antibody test and cerebrospinal fluid test findings were unremarkable. Diffusion-weighted and fluid-attenuated inversion recovery MRI of the brain showed a high signal intensity lesion at the midline of the splenium of the corpus callosum compatible with cytotoxic lesions of the corpus callosum (CLOCCs). High-dose intravenous methylprednisolone improved their symptoms and imaging findings. CLOCCs should be considered in patients with neurological manifestation after COVID-19 vaccination.

## Introduction

Various neurological adverse events can occur after coronavirus disease 2019 (COVID-19) vaccination. According to a prior study of healthcare workers, 98.34% of those who received the mRNA-1273 vaccination (Moderna) had unpleasant symptoms and 61.2% of them had trouble doing everyday activities [1]. As for the BNT162b2 vaccine (Pfizer-BioNTech), 20.3% of the recipients had troubles in activities of daily living [2]. Headache, dizziness, decreased appetite, muscle spasm, decreased sleep quality, and brain fogging were the most commonly reported symptoms relevant to the central nervous system [1, 2]. The etiology of adverse reaction to COVID-19 vaccines has not been fully elucidated. Though these symptoms are not life-threatening, they have significant impact on social unrest over unknown effect leading to negative attitudes towards vaccines and an unwillingness to receive vaccination.

Various clinical conditions are associated with cytotoxic lesions of the corpus callosum (CLOCCs) [3]. In the early stages, CLOCCs were described in patients with epileptic seizures treated with antiepileptic medications [4]. The condition was named as mild encephalitis/encephalopathy with a reversible splenial lesion (MERS) in 2004, characterized by an ovoid lesion at the midline of the splenium of the corpus callosum in patients without preceding epilepsy or viral infection as well [5]. Neurological manifestations of MERS include confusion, delirium, and seizures. Usually, they were considered as a relatively mild syndrome that resolved within a month [5–7]. Then, cumulative data revealed that the splenic lesion can be caused by a variety of diseases and conditions, some of which have a terrible prognosis [3, 8]. These lesions have been called by different names including “MERS”, “reversible splenial lesion syndrome”, “reversible splenial lesions”, “transient splenial lesions”, “clinically silent lesions in the splenium of the corpus callosum,” and “transient focal lesions in the splenium of the corpus callosum.” Recently, Starkey et al. termed these lesions as CLOCCs because restricted diffusion with low apparent diffusion coefficient (ADC) value indicates cytotoxic edema [3].

Previous reports demonstrated Japanese children with the splenic lesion after mumps vaccination [9]. In this study, we investigated the clinical features of the two cases with CLOCCs presenting neurological symptoms after receiving COVID-19 vaccination.

## Case presentation

Two adult patients, who developed neurological symptoms approximately 1 week after receipt of the first standard dose (0.3 mL, intramuscular injection) of COVID-19 mRNA vaccination (COMIRNATY intramuscular injection, Pfizer-BioNTech) (Day 1) and deteriorated over the next week, were included. Clinical course, laboratory, and MRI findings were serially analyzed. This study was carried out in accordance with the Declaration of Helsinki and was exempted from institutional ethics committee approval. Written informed consent was obtained from the patients for the publication of the case report and any accompanying images.

### Patient 1 

**Fig. 1:**
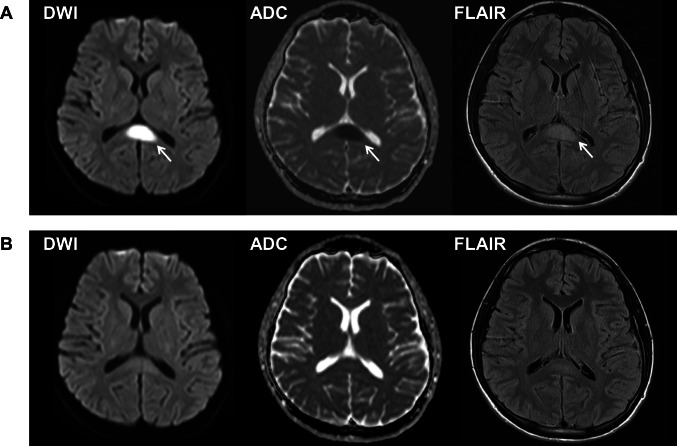
Magnetic resonance images of the brain of Patient 1 on Day 18 (**A**) and on Day 25 (**B**) after COVID-19 vaccination. **A** Diffusion-weighted image (DWI) (left) shows restricted diffusion in the splenium with low apparent diffusion coefficient (ADC) values (middle), and fluid-attenuated inversion recovery image (FLAIR) (right) shows a high signal intensity lesion at the midline of the splenium of the corpus callosum (arrows). **B** The lesion disappeared after intravenous high-dose methylprednisolone therapy

A 23-year-old man presented with nausea, mild headache, and low-grade fever (37.5 °C) (Day 9). He had no previous history of major illness and took no medication. He was a never-smoker. Neurological examination, complete blood cell count, blood chemistry including electrolytes and antibody titers, and brain magnetic resonance imaging (MRI) were unremarkable. Blood coagulation system including D-dimer was not examined. Reverse transcription polymerase chain reaction test (PCR) for severe acute respiratory syndrome coronavirus 2 (SARS-CoV-2) was negative. Cerebrospinal fluid (CSF) test showed pleocytosis (102 cells/µL (monocyte count 12 cells/µL, polycyte count 90 cells/µL), normal ≤ 5 cells/µL). Under the suspicion of aseptic meningitis, he was given intravenous acyclovir (750 mg/day) and meropenem (6.0 g/day). However, his body temperature increased up to 40 °C and he became confused, disoriented, and amnestic on Day 14. CSF test on Day 16 showed increased number of cells (942 cells/µL (monocyte count 126 cells/µL, polycyte count 816 cells/µL)) and elevated protein levels (181 mg/dL, normal 10–40 mg/dL). Interleukin (IL)-6 was markedly elevated in the CSF on Day 16 (429.0 pg/mL, cut-off value 4.3 pg/mL [10]) but normal in the serum on Day 17 (5.4 pg/mL, normal ≤ 7 pg/mL). Brain MRI on Day 18 showed an ovoid restricted diffusion in diffusion weighted imaging (DWI) in the splenium with low apparent diffusion coefficient (ADC) values (Fig. 1). Contrast-enhanced MRI was not obtained. Based on the typical imaging features, he was diagnosed with CLOCCs. Additional therapy with intravenous high-dose methylprednisolone (2 courses of 1,000 mg/day for 3 days; Day 18–Day 20 and Day 24–Day 26) was effective. On Day 25, the splenial lesion disappeared (Fig. 1). He completely recovered on Day 26, and afterwards, no residual symptoms or recurrence was found during follow-up period of 8 months.

### Patient 2

**Fig. 2:**
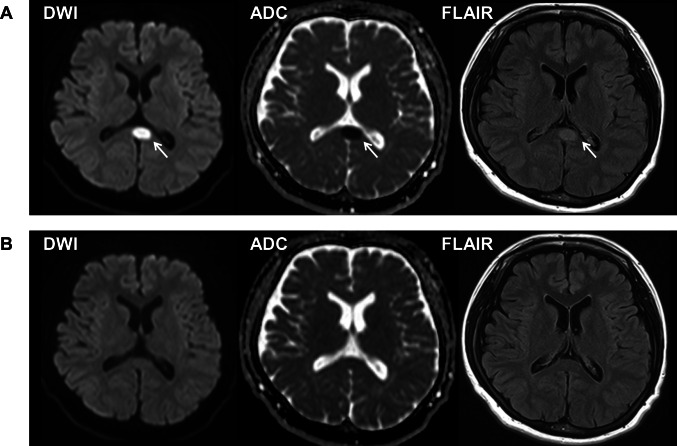
Magnetic resonance images of the brain of Patient 2 on Day 17 (**A**) and on Day 29 (**B**) after COVID-19 vaccination. **A** Diffusion-weighted image (DWI) (left) shows restricted diffusion in the splenium with low apparent diffusion coefficient (ADC) values (middle), and fluid-attenuated inversion recovery image (FLAIR) (right) shows a high signal intensity lesion at the midline of the splenium of the corpus callosum (arrows). **B** The lesion disappeared after intravenous high-dose methylprednisolone therapy

A 33-year-old woman with a history of mild mental retardation and depression presented with visual disturbance and headache lasting for several days (Day 11). She was taking no medication and smoked 3 cigarettes per day on average. PCR for SARS-CoV-2 was negative. She was afebrile, and general physical examination was unremarkable. Neurological examination revealed dysarthria, a left forearm tremor, dysesthesia of the mouth and distal limbs, visual agnosia, and metamorphopsia. Complete blood cell count and blood chemistry including electrolytes and antibody titers were unremarkable. Blood coagulation system including D-dimer was not examined. Brain MRI on Day 17 showed a lesion in the splenium compatible to CLOCCs (Fig. 2). Contrast-enhanced MRI was not obtained. Serum IL-6 was not elevated on Day 17 (2.5 pg/mL). CSF test was not performed. Meanwhile, electroencephalography revealed slow waves in the left posterio-temporo-occipital areas. Intravenous high-dose methylprednisolone therapy (1 course of 1,000 mg/day for 3 days; Days 18–20) was effective. The splenial lesion fully improved on Day 29 (Fig. 2). Her neurological symptoms gradually recovered over the next month. Thereafter, no recurrence occurred during follow-up period of 7 month.

## Discussion

To the best of our knowledge, this is the first report of neurological manifestations with CLOCCs that occurred approximately 1 week after COVID-19 vaccination. Thus far, 2 case reports showed that CLOCCs emerged 2 or 3 days after COVID-19 vaccination [11, 12]. On the other hand, Patient 1 in our study demonstrated that MRI on Day 9 was negative for the splenial lesion and CLOCCs was noticed on Day 18, and IL-6 elevation in the CSF. Clinical course of both Patients 1 and 2 showed subacute progression over weeks and effectiveness of immunotherapy. The findings shed light on possible role of delayed immune response with cytokine storm in long-lasting neurological symptoms associated with COVID-19 vaccination and even with COVID-19 itself, and need for immunomodulation and immunosuppression therapies.

An ovoid lesion in the midline of the splenium, not accompanied by any other imaging abnormality, has been associated with preexisting epilepsy under antiepileptic treatment and viral infection. Tada et al. investigated clinical features of 15 patients with new onset encephalitis/encephalopathy with reversible splenial lesion and reported that the patients showed relatively mild neurological manifestations and completely recovered in 1 month [5]. As per the national survey of pediatric acute encephalopathy in Japan, CLOCCs is the second most frequent syndrome (215 cases, 19.3%), and is most commonly associated with influenza virus [13]. Besides intracranial direct viral infection, hemodynamic, metabolic, electrolyte, or autoimmunity are possible mechanism of CLOCCs.

CLOCCs after vaccination are relatively uncommon. Only five male youngsters have developed CLOCCs after mumps immunization so far [9]. All 5 patients showed hyponatremia, and mumps vaccine virus (Hoshino strain and Torii strain) was confirmed in their CSF. In contrast, hyponatremia or other electrolyte imbalances were not detected in our patients. SARS-CoV-2 infection was also unlikely based on negative PCR result. Recently, increased cytokines/chemokines were demonstrated in patients with MERS associated with acute focal bacterial nephritis, which were normalized in several weeks [14]. In the current study, Patient 1 showed marked elevation of CSF IL-6 level according to cut-off value of lupus psychosis [10]. Therefore, possible role of cytokine storm in neurological symptoms associated with COVID-19 vaccination and even with COVID-19 itself should be further elucidated [15]. Information on other cytokines such as IL-8, IL-1α, and tumor necrosis factor-α would be helpful to investigate autoimmune mechanism in the central nervous system. Serum level of IL-6 was within normal limit in both of our patients; thus, CSF test would be necessary. Contrast-enhanced MRI is useful to exclude acute disseminated encephalomyelitis though sole involvement of corpus callosum is rare [16].

Though neurological manifestations of CLOCCs are mild and can be reversible, patients with overt disturbance or alteration of consciousness, focal neurological deficits, or seizures, especially with progression as in our cases, are treated with steroids and immunoglobulin [3, 5, 9, 13, 14].

## Conclusions

In conclusion, CLOCCs should be considered in patients who had neurological symptoms after receiving COVID-19 vaccination, even if the symptoms were mild and nonspecific.

## Data Availability

The data that support the findings of this study are available on request from the first author (H. O.). The data are not publicly available due to information that could compromise research participant privacy.

## Abbreviations

COVID-19: Coronavirus disease 2019
CLOCCs: Cytotoxic lesions of the corpus callosum
MERS: Mild encephalitis/encephalopathy with a reversible splenial lesion
MRI: Magnetic resonance imaging
DWI: Diffusion weighted imaging
ADC: Apparent diffusion coefficient
PCR: Reverse transcription polymerase chain reaction test
SARS-CoV-2: Severe acute respiratory syndrome coronavirus 2
CSF: Cerebrospinal fluid
IL: Interleukin
